# Rapid syllable transition treatment (*ReST*) in children with speech motor delay: case studies

**DOI:** 10.1590/2317-1782/e20250363en

**Published:** 2026-08-17

**Authors:** Ana Carolina Bucci, Camila Goulart, Aline Mara de Oliveira

**Affiliations:** 1 Clínica Fonoaudiológica Privada - Florianópolis (SC), Brasil.; 2 Departamento de Fonoaudiologia, Centro de Ciências da Saúde, Universidade Federal de Santa Catarina – UFSC - Florianópolis (SC), Brasil.

**Keywords:** Speech Sound Disorders, Speech Therapy, Speech Intelligibility, Speech-Language Rehabilitation, Apraxia of Speech

## Abstract

Speech Motor Delay (SMD) is characterized by difficulties in motor execution, resulting in unintelligible speech, with changes in articulatory precision, stability, voice, and prosody, as well as reduced articulatory dissociation and excessive movements for age. The objective of this study was to describe, through four longitudinal case studies, the performance of Brazilian children with SMD submitted to the Rapid Syllable Transition Treatment (ReST), verifying acquisition, retention, and generalization. Four monolingual children, aged five to eight years and diagnosed with SMD, participated. The intervention was conducted via synchronous telepractice, twice a week, over six weeks. Probes of trained and untrained pseudowords, sentences, real words, and control items were applied at different stages of therapy. The results showed progressive improvement in all evaluated aspects. Significant gains were observed in phoneme accuracy, lexical stress, and coarticulation, confirming acquisition, retention, and generalization. It is concluded that ReST is effective for children with SMD, including when applied through telepractice, representing a promising evidence-based therapeutic alternative grounded in motor learning principles and supported by linguistically controlled stimuli selection.

## INTRODUCTION

Speech Sound Disorders (SSDs) encompass any difficulty, or combination of difficulties, related to the perception, motor production, or phonological representation of speech sounds and segments, and may be classified as either organic or functional in nature. Organic SSDs arise from underlying motor/neurological, structural, or sensory-perceptual impairments, whereas functional SSDs are considered idiopathic, that is, they do not present an identifiable underlying cause^(1)^.

SSDs are classified into three major categories of speech disorders: Speech Delay (previously referred to as phonological disorder), Residual Speech Errors, and Motor Speech Disorders^(2)^.

Speech Motor Delay (SMD) is included within the classification of Motor Speech Disorders (MSDs), alongside Childhood Apraxia of Speech (CAS), Childhood Dysarthria (CD), and the comorbid presentation of CAS + CD. These four classifications may occur in association with either idiopathic speech disorders or complex neurodevelopmental disorders^(3,4)^.

SMD, previously referred to as Non-Specific Motor Speech Disorder or Speech Sound Disorder with a Motor Component, does not meet the diagnostic criteria for CD or CAS. Nevertheless, it is characterized by impairments in neuromotor execution, reflecting a delay in the maturation of the speech motor system. Such impairments may result in deficits in articulatory precision, speech stability, voice quality, and prosody, in addition to the possible presence of reduced dissociation of speech movements and excessively broad movement amplitudes that are not developmentally expected for the individual’s age^(3,5,6)^.

For the diagnosis of SMD, one diagnostic approach involves identifying the presence of at least four out of nine speech motor markers, also referred to as red flags. These markers include: lateral jaw sliding; reduced lip retraction and rounding movements; inadequate coordination between the jaw and lips across two planes of movement; difficulty dissociating tongue-tip movements from jaw movements; difficulty alternating the place of articulation; reliance on jaw movements to drive speech production; limited production of vowels and consonants, accompanied by vowel and consonant distortions; restricted syllabic forms and word structures; and disrupted speech production as sentence length and complexity increase^(6)^.

During therapeutic intervention for cases of MSDs, intervention approaches grounded in the principles of motor learning are strongly recommended, given that speech production itself constitutes a motor skill. Consequently, such approaches may substantially contribute to improvements in both speech acquisition and the organization of the speech motor system. Furthermore, the current literature indicates that children with MSDs tend to demonstrate limited responsiveness to traditional phonological and articulatory intervention approaches^(6,7)^.

Currently, the intervention approaches for MSDs that are supported by scientific evidence and grounded in the principles of motor learning include: Ultrasound Biofeedback for Tongue Movement^(8)^; Dynamic Temporal and Tactile Cueing (DTTC)^(9)^; the Nuffield Dyspraxia Programme, Third Edition (NDP3)^(10,11)^; Prompts for Restructuring Oral Muscular Phonetic Targets (PROMPT)^(6)^; and Rapid Syllable Transition Treatment (ReST)^(9,10)^.

ReST is an **evidence-based therapeutic intervention model with documented clinical applicability**, primarily designed for the treatment of children with MSDs, particularly CAS. However, current evidence in the literature has begun to suggest its **potential applicability to other MSD profiles, including SMD** ^(12,13)^.

The model was developed by researchers at the University of Sydney with the aim of directly addressing the speech-related motor planning and programming deficits underlying these disorders. Subsequently, it was adapted into several other languages, including Arabic, Cantonese, Danish, Dutch, French, German, Italian, Korean, Spanish, Swedish, Tagalog, English, and Portuguese^(12,14,15)^.

The translation and adaptation of this intervention model into Brazilian Portuguese were undertaken with the aim of promoting its broader applicability in both face-to-face clinical settings and telepractice. This initiative is particularly relevant given the current scarcity, in Brazil, of scientifically grounded studies involving children with MSDs that are informed by linguistic and cultural criteria^(13,14,16)^.

The ReST intervention model consists of high-intensity practice using pseudowords, as their use enables children to rehearse speech motor planning and programming in a manner analogous to real-word production, without interference from previously acquired and incorrectly established motor speech patterns. **Furthermore, the use of pseudowords allows for greater linguistic control over the stimuli (e.g., syllabic structure and stress pattern), thereby enhancing the comparability and interpretation of outcomes.** The pseudowords are presented in a randomized manner across 12 treatment sessions, with variations in phonetic structure and lexical stress. Additionally, the sessions are divided into two phases: pre-practice (also referred to as the training phase) and practice, both of which are designed to incorporate and maximize the application of motor learning principles^(17)^.

Currently, ReST therapy is recommended for children aged five years and older who have been diagnosed with CAS, with severity ranging from mild to severe. Initially, the level of pseudowords to be selected for treatment is determined, and, if the child demonstrates the necessary prerequisites, intervention is initiated using disyllabic pseudowords, trisyllabic pseudowords, or Cloze sentences^(17)^.

Throughout the implementation of the ReST intervention approach, probe assessments are administered prior to the onset of treatment and at the beginning of the 4th, 8th, and 12th sessions in order to evaluate acquisition, retention, and generalization **across the different probe categories**, namely: treated pseudowords, untreated pseudowords, Cloze sentences containing trisyllabic pseudowords, real words, and control items^(17)^.

## PRESENTATION OF THE CLINICAL CASES

This study is linked to the research project *Phonological Disorder and Apraxia Outpatient Clinic*, approved by the Research Ethics Committee of Federal University of Santa Catarina (Approval No. 35360620.9.0000.0121). All participants and their legal guardians were informed about the study and, upon agreeing to participate, signed the Informed Consent Form (ICF), the Assent Form, and the Ethics Committee Approval Document.

Four Brazilian monolingual children aged between five and eight years participated in the study, all of whom were diagnosed with SMD. **A four-case study design was adopted to document individual variability in response to ReST, enabling the observation of patterns of acquisition, retention, and generalization over time within an exploratory longitudinal design.** This approach is particularly appropriate given that it represents an initial and pioneering investigation within the Brazilian context. The intervention was conducted using the Rapid Syllable Transition Treatment (ReST) method, delivered via synchronous telepractice, with a frequency of two sessions per week over a six-week period. **Telepractice was chosen to improve access to therapy and support treatment continuity, as well as to align with recent evidence and recommendations indicating the feasibility of speech motor interventions delivered remotely, provided that structure, dosage, and feedback control are maintained.**

The inclusion criteria were as follows: signed informed consent form (ICF) by the legal guardians; being a native speaker of Brazilian Portuguese (BP); absence of associated comorbidities, verified through detailed clinical anamnesis and information provided by parents; receptive language performance superior to expressive language performance, confirmed through clinical observation and simple comprehension tasks; hearing thresholds within normal limits; preserved visual acuity; and age between four and ten years.

Data collection was organized into three main stages. The first stage included anamnesis and speech-language assessment, conducted using the Orofacial Praxis Test^(18)^ and the Child Phonological Assessment (CPA)^(19)^. The second stage involved diagnostic speech motor assessment, which included the Inconsistency Speech Test^(20)^, the Multisyllabic Word Repetition Task^(20)^, and the Lexical Sentence Task^(20)^. The third stage consisted of the therapeutic intervention using the ReST method.

For the intervention, four vowels and four consonants were selected as the basis for constructing a list of 20 pseudowords. These pseudowords, either disyllabic or trisyllabic, were designed according to each child’s level of difficulty, following the protocol proposed by Oliveira and Oliveira^(20)^, which includes variation in lexical stress placement. **It is noteworthy that the selection of pseudowords followed carefully defined linguistic criteria (control of syllabic structure and stress pattern), as recommended for the ReST protocol, thereby enhancing interpretability and replicability.** The sessions were structured in two phases: a pre-practice phase, in which the clinician provided greater support through immediate feedback and modeling, and a practice phase, in which pseudowords were practiced in blocks with delayed, summary, and randomized feedback, interspersed with short rest intervals.

Probes were administered at different time points: prior to the onset of therapy, after the 4th and 8th sessions, at the end of the 12th session, and subsequently at a follow-up assessment four months after the completion of the intervention. At each probe session, trained and untrained pseudowords, sentences, real words, and control items were assessed in order to examine acquisition, retention, and generalization. **Families were instructed to support the implementation of the sessions and to maintain, throughout the week, an environment conducive to practice and routine stability, without replacing the structured therapeutic training outlined in the protocol.**

**Figure 1:**
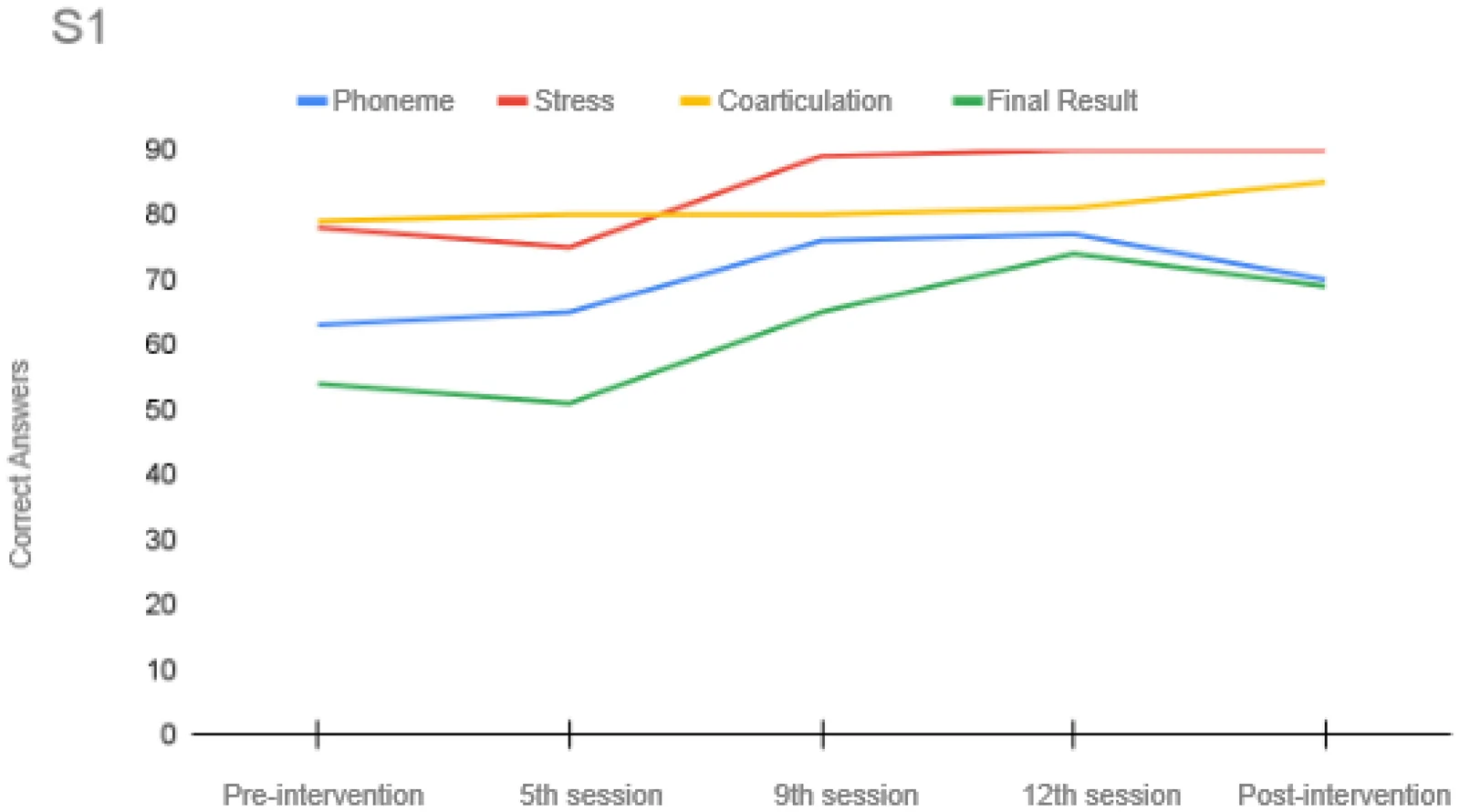
Graph illustrating S1’s performance in phoneme accuracy, lexical stress, coarticulation, and overall production outcome across the probe assessments conducted throughout the study

**Source:** Prepared by the authors

**Figure 2 gf0200:**
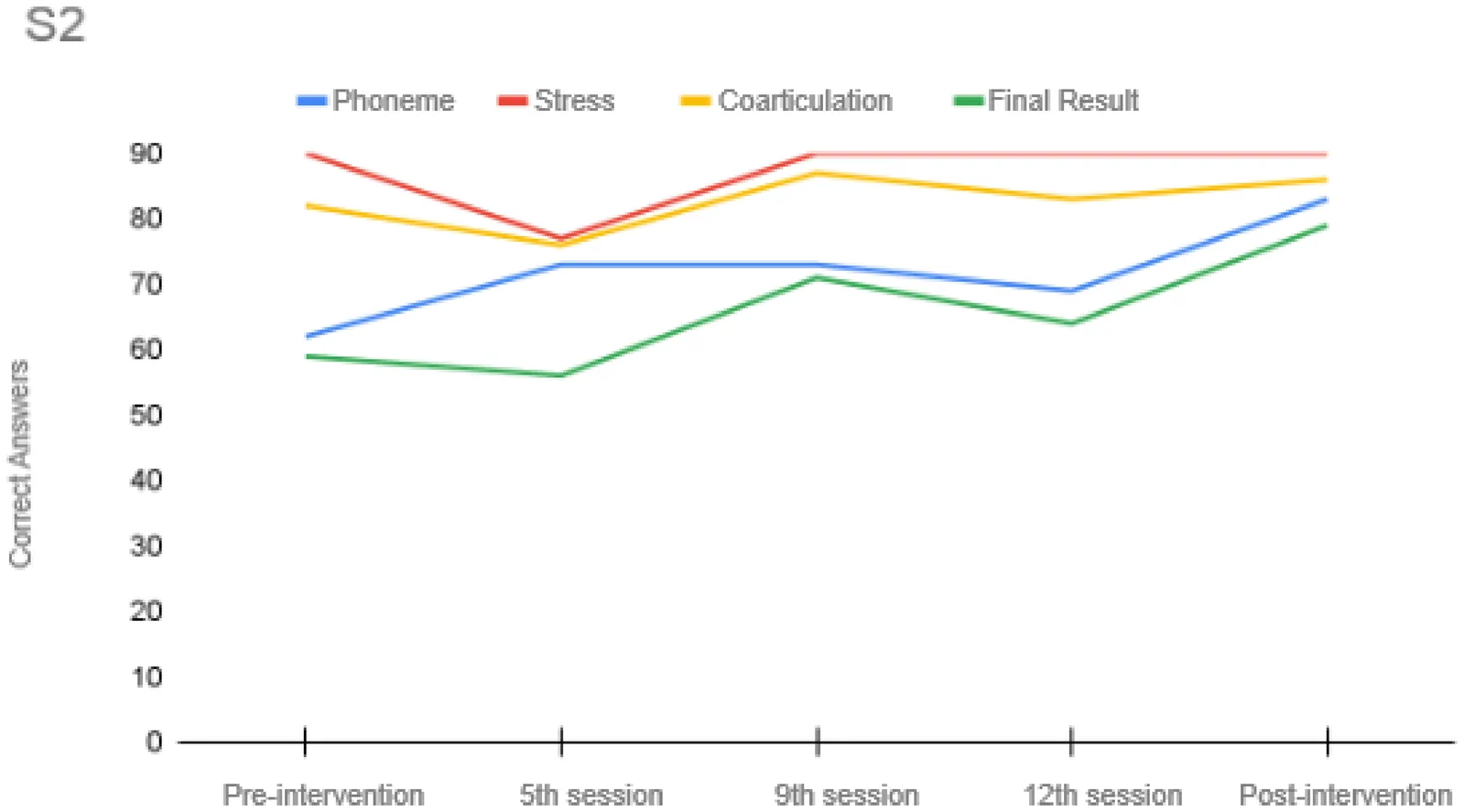
Graph illustrating S2’s performance in phoneme accuracy, lexical stress, coarticulation, and overall production outcome across the probe assessments conducted throughout the study

**Source:** Prepared by the authors

**Figure 3 gf0300:**
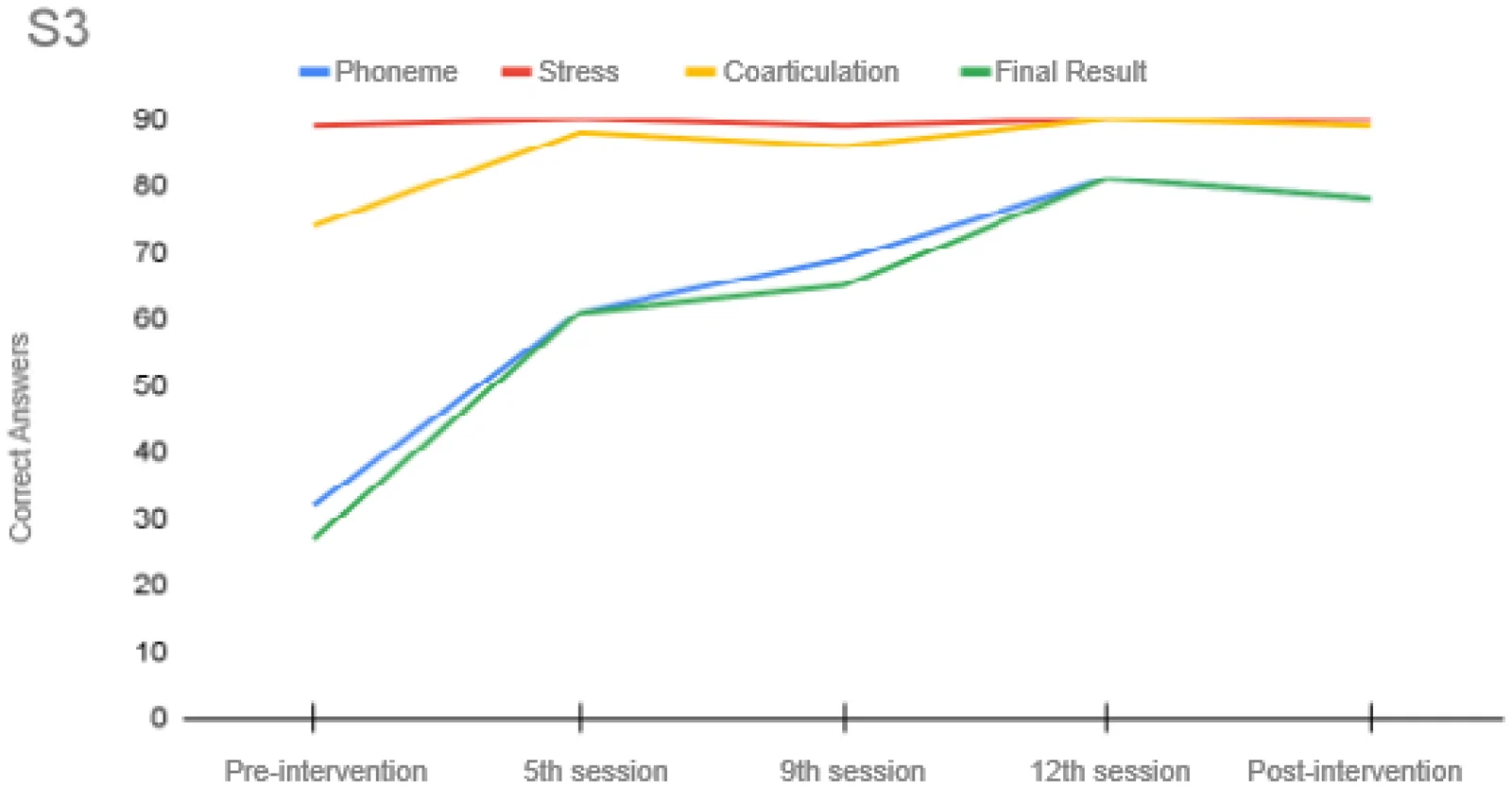
Graph illustrating S3’s performance in phoneme accuracy, lexical stress, coarticulation, and overall production outcome across the probe assessments conducted throughout the study

**Source:** Prepared by the authors

**Figure 4:**
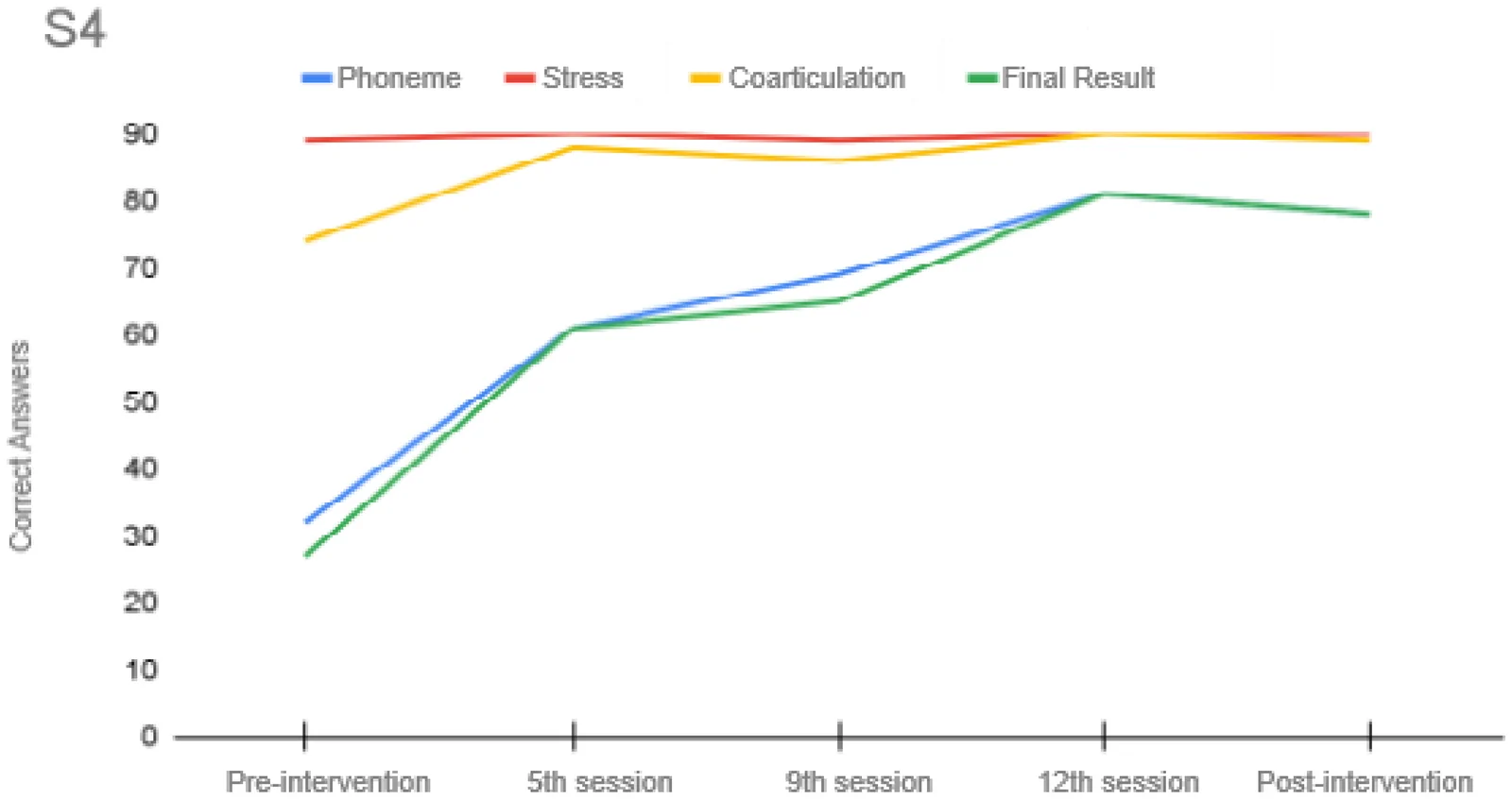
Graph illustrating S4’s performance in phoneme accuracy, lexical stress, coarticulation, and overall production outcome across the probe assessments conducted throughout the study

**Source:** Prepared by the authors

Individual results are presented quantitatively in Tables 1, 2, and 3 and are illustrated graphically in Figures 1 to 4.

**Table 1:** Nominal qualitative data presenting the clinical profile of each study participant

	**AGE RANGE**	**SEX**	**REGION**	**SEVERITY**
S1	5 years old	Male	Southeast	Moderately severe
S2	7 years old	Male	South	Moderately severe
S3	7 years old	Male	South	Mild-to-moderate
S4	8 years old	Male	Southeast	Mild-to-moderate

**Caption:** S1 = Participant 1; S2 = Participant 2; S3 = Participant 3; S4 = Participant 4. Source: Prepared by the authors

**Table 2:** Continuous quantitative data presenting the PCC index before and after the ReST intervention for each study participant

**PARTICIPANTS**	**PRE-INTERVENTION PCC**	**POST-INTERVENTION PCC**
S1	61.95%	82.30%
S2	55.77%	69.75%
S3	80.22%	81.25%
S4	79.25%	79.71%

**Caption:** S1 = Participant 1; S2 = Participant 2; S3 = Participant 3; S4 = Participant 4. Source: Prepared by the authors

**Table 3:** Mean, median, minimum, and maximum values obtained from the probe assessments conducted with all study participants

**VARIABLE**	**MEAN (SD)**	**MEDIAN**	**MIN.–MAX.**
**Trained pseudowords (20)**			
Pre-intervention	12.75 (4.26)	13	8-17
5th session	15.25 (4.97)	17	7-20
9th session	18 (0.71)	18	17-19
12th session	18 (1.22)	17.5	17-20
Delayed post-treatment assessment	17.5 (2.18)	18	14-20
**Untrained pseudowords (20)**			
Pre-intervention	7.5 (6.22)	6	1-17
5th session	12.75 (2.28)	13.5	9-15
9th session	15.5 (0.87)	15	15-17
12th session	17 (1.73)	16	16-20
Delayed post-treatment assessment	15 (2.24)	15	12-18
**Sentences (20)**			
Pre-intervention	6.75 (476)	5.5	2-14
5th session	11.75 (2.49)	12.5	8-14
9th session	14.25 (3.03)	14.5	10-18
12th session	16.5 (2.50)	16.5	13-20
Delayed post-treatment assessment	13.75 (2.28)	14	11-16
**Real words (20)**			
Pre-intervention	14.25 (3.34)	14.5	10-18
5th session	16.75 (1.79)	17	14-19
9th session	16 (2.45)	17	12-18
12th session	17.25 (2.28)	17	15-20
Delayed post-treatment assessment	18.5 (2.06)	19.5	15-20
**Real words involving different sound processes (10)**			
Pre-intervention	3.25 (2.77)	2	1-8
5th session	3.75 (3.11)	2.5	1-9
9th session	2.75 (3.63)	1	0-9
12th session	4.75 (4.32)	4.5	0-10
Delayed post-treatment assessment	7.25 (2.77)	7.5	4-10

Mann–Whitney Test

**Caption:** SD = Standard deviation; Min. = Minimum value; Max. = Maximum value. Source: Prepared by the authors

Each figure corresponds to the performance of a single participant, allowing for the visualization of their progress in terms of phoneme accuracy, lexical stress, coarticulation, and overall production outcomes. For instance, Figure 1 illustrates the progression of participant S1 across probe assessments, with a gradual increase in articulatory accuracy; Figure 2 shows similar results for S2; whereas Figures 3 and 4 depict the performance of participants S3 and S4, respectively, both of whom also demonstrated consistent improvement, albeit at different rates. All stages were conducted by the same speech-language pathologist, ensuring consistency in the therapeutic process **(which supports standardization), although this may introduce assessment bias, as discussed below.**

## DISCUSSION

The findings of this case study indicate systematic improvements in speech performance following the ReST intervention, as reflected by increased Percentage of Consonants Correct (PCC)^(21)^ scores (Table 2) and by the progression observed across probes involving trained and untrained pseudowords, sentences, and real words (Table 3). Individual analyses (Figures 1 [Fig gf0200] [Fig gf0300]-4) revealed a consistent trajectory of improvement in phoneme accuracy, lexical stress, and coarticulation, with a direct impact on overall speech production outcomes. Although participants S3 and S4 remained within the same severity classification according to Shriberg et al.^(21)^, the observed trend toward percentage gains suggests clinically meaningful benefits and proximity to a threshold of functionally relevant change.

**The interpretation of these findings is consistent with the literature supporting ReST** as an intervention grounded in motor learning principles (variability, distributed practice, random sequencing, and delayed feedback management), which are known to promote retention and generalization^(7,12)^. The use of pseudowords may have mitigated interference from previously stabilized motor programs, thereby enabling the reorganization of motor plans and facilitating both prosodic (lexical stress) and segmental (phoneme accuracy) control, which was reflected in the progressive improvements observed across the intermediate probes and follow-up assessment^(15-17)^ *.*

Comparatively, our findings are consistent with those reported by Oliveira and Oliveira^(14)^, who documented acquisition and generalization gains in Brazilian children with CAS undergoing ReST intervention, including through telepractice delivery. **Furthermore, the literature has described favorable outcomes associated with ReST in face-to-face clinical settings and has increasingly discussed the feasibility of telepractice for MSDs, provided that minimum standards of quality and procedural standardization are ensured.** Although the treatment dosage employed in the present study (two sessions per week over six weeks) was less intensive than high-frequency protocols, the direction and magnitude of the observed gains were consistent with the pattern of improvement described in the literature, particularly with respect to the increases observed in untrained items and real words (Table 3). This pattern is also consistent with the analysis proposed by Bucci^(13)^ regarding treatment intensity and dosage, suggesting that lower-frequency intervention formats may still be effective when the critical practice and feedback components of ReST are preserved.

**A clinically relevant finding was the pattern of delayed generalization observed for real words involving different sound processes (Table 3): scores were more modest during the intermediate probes and increased at the delayed post-treatment assessment.** This delayed pattern is plausible in SMD, in which the stabilization of new motor plans requires greater time and broader exposure to varied phonotactic contexts. In other words, transfer to less familiar phonological and prosodic targets tends to emerge only after more fundamental motor control has been consolidated. From a therapeutic perspective, these findings reinforce the importance of maintaining contextual and prosodic variability in practice even after the completion of the formal treatment block, thereby promoting extended retention. **Additionally, these results support the recommendation for follow-up periods longer than four months in order to capture trajectories of delayed consolidation and maintenance in naturalistic contexts (e.g., home and school environments).**

**The feasibility of telepractice constitutes another noteworthy finding. The maintenance of treatment gains within a remote setting suggests good protocol transferability, provided that key components are preserved, including session structure (pre-practice/practice), item randomization, feedback pacing, and the quality of auditory and visual cues.** However, telepractice introduces potential confounding factors, including connection quality, environmental noise, and variability in family support, all of which should be monitored and, ideally, controlled in subsequent studies. **Therefore, the adoption of minimum environmental standardization procedures and simple signal-quality metrics is recommended, along with the systematic documentation of these conditions throughout the course of treatment.**

This study also provides insights into specific mechanisms of change in SMD. The parallel increases observed in segmental accuracy and lexical stress performance (Figures 1 [Fig gf0200] [Fig gf0300]-4) suggest that temporal (prosodic) and spatial (articulatory) alignment may have been functionally co-trained through ReST, thereby reducing reliance on compensatory strategies (e.g., excessive jaw use). This finding is consistent with the hypothesis that adjustments in timing and stress hierarchy function as global organizers of articulatory gestures in children with delayed motor speech profiles.

Particular emphasis should be placed on the contribution of the qualitative analysis of outcomes in the present study. The longitudinal monitoring of individual performance (Figures 1 [Fig gf0200] [Fig gf0300]-4) allowed for the observation of participant-specific progression patterns, including gradual improvements in phonemic accuracy, lexical stress, and coarticulation. This type of analysis is particularly valuable in preliminary investigations, especially in areas that remain underexplored in Brazil, such as the application of ReST in children with SMD. By emphasizing individual clinical progression, it becomes possible to capture nuances of the therapeutic process that may not be fully reflected through statistical measures alone, thereby reinforcing the clinical applicability of the method in speech-language pathology practice.

The present study has certain limitations that should be acknowledged, including the small number of participants (n=4) and the absence of a control group, both of which restrict the generalizability of the findings. **From an exploratory perspective, no single pattern was identified indicating superior outcomes exclusively associated with either greater or lesser severity, thereby reinforcing the need for larger samples and severity stratification in future studies.** Data collection was conducted by a single speech-language pathologist, which ensured procedural consistency but may also have introduced assessment-related bias. Despite these limitations, it is important to emphasize that this is the first study to investigate the application of ReST in children with SMD in Brazil, providing relevant preliminary evidence and paving the way for future controlled studies with larger samples.

To strengthen the evidence base, future studies should adopt controlled designs (ideally randomized clinical trials) involving larger samples and **severity stratification for SMD. It is also recommended that future investigations examine potential moderators (e.g., age, severity, and prosodic profile) and mediators (e.g., treatment adherence, practice distribution, and feedback frequency) in order to clarify which individuals benefit most from ReST and under which dosage conditions. In addition to larger sample sizes, future studies should seek to standardize telepractice procedures and conditions, incorporate blinded outcome assessment, and extend follow-up periods in order to evaluate maintenance and generalization within naturalistic communicative contexts.**

Within the context of telepractice, minimum standards for environmental conditions and signal-quality metrics may reduce variability and enhance reproducibility. Finally, follow-up periods extending beyond four months may better capture trajectories of delayed consolidation and the maintenance of generalization across naturalistic communicative settings (e.g., school and home environments).

## FINAL REMARKS

This study demonstrated that the application of the ReST method in Brazilian children with SMD resulted in consistent gains in phonemic accuracy, lexical stress, and coarticulation, while also promoting the acquisition, retention, and generalization of treatment targets. These findings further support the effectiveness of the method, including when delivered through telepractice, thereby expanding the possibilities for access to intervention. **It should also be emphasized that the selection of pseudowords followed controlled linguistic criteria, a methodologically relevant aspect for the interpretation of the findings.**

**Despite its methodological limitations, this is the first Brazilian study to systematically investigate the application of ReST in children with SMD,** thereby conferring a pioneering character and clinical relevance to the findings. The qualitative analysis of the data, together with the progression observed across probe assessments, provides preliminary evidence that ReST may be incorporated as a promising therapeutic approach within speech-language pathology.

Future studies involving larger samples and more robust methodological designs may further confirm and extend these findings; nevertheless, the present data already contribute to informing clinical practice and strengthening the national literature in the field of MSDs. **Subsequent investigations employing more rigorous designs—including controlled protocols, severity stratification, blinded assessors, standardized telepractice procedures, and longer follow-up periods—may further expand and consolidate the evidence presented herein.**
