# Supplementary material for: Rapid syllable transition treatment (ReST) in children with speech motor delay: case studies
Source: Codas. 2026 Aug 17;38(5):e20250363. doi: 10.1590/2317-1782/e20250363en (PMC13520640; doi:10.1590/2317-1782/e20250363en)
Supplement: Translation

# Translation: Rapid syllable transition treatment (*ReST*) em crianças com atraso motor de fala: estudos de casos

**DOI:** 10.1590/2317-1782/e20250363pt

**Authors:** Ana Carolina Bucci, Camila Goulart, Aline Mara de Oliveira

**Affiliations:** 1 Clínica Fonoaudiológica Privada - Florianópolis (SC), Brasil.; 2 Departamento de Fonoaudiologia, Centro de Ciências da Saúde, Universidade Federal de Santa Catarina – UFSC - Florianópolis (SC), Brasil.

**Keywords:** Transtornos dos Sons da Fala, Terapia da Fala, Inteligibilidade da Fala, Reabilitação dos Transtornos da Fala e da Linguagem, Apraxia da Fala

## Abstract

O Atraso Motor de Fala (AMF) caracteriza-se por dificuldade na execução motora, resultando em fala ininteligível, com alterações na precisão articulatória, estabilidade, voz e prosódia, além de dificuldades na dissociação dos articuladores e na presença de movimentos excessivos para a idade. O objetivo deste estudo foi descrever, por meio de quatro estudos de caso longitudinais, o desempenho de crianças brasileiras com AMF submetidas ao Rapid Syllable Transition Treatment (ReST), verificando aquisição, retenção e generalização. Participaram quatro crianças monolíngues, entre cinco e oito anos, diagnosticadas com AMF. A intervenção foi realizada por teleatendimento síncrono, duas vezes por semana, durante seis semanas. Foram aplicadas sondagens em diferentes momentos da terapia, envolvendo pseudopalavras treinadas e não treinadas, frases, palavras reais e itens de controle. Os resultados mostraram aumento progressivo da pontuação em todos os aspectos avaliados. Observou-se melhora significativa na acurácia dos fonemas, no acento lexical e na coarticulação, confirmando aquisição, retenção e generalização. Conclui-se que o ReST mostrou-se um método promissor para crianças com AMF, inclusive em formato de teleatendimento, configurando-se como alternativa terapêutica promissora fundamentada nos princípios da aprendizagem motora e em critérios linguísticos na seleção de estímulos.

## INTRODUÇÃO

Os Transtornos dos Sons da Fala (TSFs) referem-se a qualquer dificuldade ou combinação de dificuldades em relação a percepção, produção motora ou representação fonológica dos sons e segmentos da fala, podendo ser de natureza orgânica ou funcional. Os TSFs orgânicos resultam de uma causa motora/neurológica, estrutural ou sensorial/perceptual subjacente, já os TSFs funcionais são idiopáticos, ou seja, não apresentam causa conhecida^(1)^.

Os TSFs são classificados em três tipos principais de desordens da fala, sendo eles o Atraso de Fala (anteriormente denominado de desvio fonológico), Erros Residuais de Fala e Transtornos Motores de Fala^(2)^.

O Atraso Motor de Fala (AMF) está incluído na classificação dos Transtornos Motores de Fala (TMF), juntamente com Apraxia de Fala na Infância (AFI), Disartria Infantil (DI) e AFI + DI em comorbidade. As quatro classificações podem ocorrer, com distúrbios idiopáticos de fala ou transtornos complexos do neurodesenvolvimento^(3,4)^.

O AMF, anteriormente chamado de Transtorno Motor de Fala Não-Especificado ou de Transtorno de Som de Fala com Componente Motor, não atende aos critérios para o diagnóstico de DI e AFI, sendo o mesmo o causador de desordens na execução neuromotora, ou seja, ocorre atraso na maturação do sistema motor da fala, provocando alterações na precisão articulatória, na estabilidade da fala, na voz e na prosódia, além de poder apresentar conjuntamente falta de dissociação dos movimentos da fala e da amplitude de movimentos excessivos, não esperados para a idade^(3,5,6)^.

Para o diagnóstico de AMF, uma das formas de análise diagnóstica é que a criança apresente no mínimo quatro de nove marcadores motores de fala, também denominados de bandeiras vermelhas, sendo eles: deslizamento lateral da mandíbula; movimentos reduzidos de retração e arredondamento dos lábios; coordenação inadequada da mandíbula e dos lábios em dois planos de movimento; dificuldade para dissociar a ponta da língua da mandíbula; dificuldade em alternar o local da articulação; usar a mandíbula para conduzir movimentos da fala; poucas produções de vogais e consoantes, além de distorcer as vogais e consoantes; poucas formas silábicas e estruturas de palavras; fala interrompida quando a extensão e a complexidade das sentenças aumentam^(6)^.

Durante a intervenção terapêutica dos casos de TMF, são recomendados métodos de intervenção baseados nos princípios da aprendizagem motora, tendo em vista que a produção da fala também é uma habilidade motora, portanto, este método pode auxiliar grandemente na melhora do indivíduo, tanto na aprendizagem quanto na organização do sistema motor da fala. Também se deve considerar que a literatura atual relata que crianças com TMF são resistentes às abordagens tradicionais de intervenção fonológica e de articulação^(6,7)^.

Na atualidade, os métodos de intervenção para os TMF que possuem evidências científicas e são baseados nos princípios da aprendizagem motora, são: *Biofeedback Ultrassonografia de língua* ^(8)^; *Dynamic Temporal and Tactile and Cueing (DTTC)* ^(9)^; *Nuffield Dyspraxia Program, Third Edition (NDP3)* ^(10,11)^ *; Prompts for Restructuring Oral Muscular Phonetic Targets (PROMPT)* ^(6)^; *Rapid Syllable Transition Treatment (ReST)* ^(9,10)^ *.*

*ReST* é um modelo de intervenção terapêutica, **baseado em evidências científicas e com aplicabilidade documentada**, e focado no tratamento de crianças com TMF, especificamente AFI, porém atualmente já existem indícios na literatura **de sua aplicabilidade em outros perfis de TMF, incluindo o AMF** ^(12,13)^.

O modelo foi desenvolvido com o objetivo de abordar diretamente os problemas de planejamento e de programação motora subjacentes à fala, por pesquisadores da Universidade de Sydney, e posteriormente, foi adaptado para outras línguas como Árabe, Cantonês, Dinamarquês, Holandês, Francês, Alemão, Italiano, Coreano, Espanhol, Sueco, Tagalo, Inglês e Português^(12,14,15)^.

A tradução e adaptação do modelo de intervenção para o Português Brasileiro, foi realizada objetivando contribuir para a popularização da aplicabilidade no setting clínico presencial e por meio do teleatendimento, tendo em vista que atualmente no Brasil, nota-se grande escassez de estudos com crianças que possuem TMF baseados em evidências científicas e pautados em critérios linguísticos e culturais^(13,14,16)^.

O modelo de intervenção *ReST*, consiste na prática de alta intensidade de pseudopalavras, tendo em vista que o uso das mesmas permite que as crianças pratiquem o planejamento e a programação motora de forma semelhante a palavras reais, sem a interferência de planos motores aprendidos anteriormente de forma incorreta. **Além disso, as pseudopalavras possibilitam maior controle linguístico dos estímulos (por exemplo, estrutura silábica e padrão acentual), favorecendo comparabilidade e interpretação dos resultados.** As pseudopalavras são apresentadas aleatoriamente, durante 12 sessões, com variações na estrutura fonética e no acento lexical, além disso, as sessões são divididas em duas etapas, sendo elas: pré-prática (também podendo ser denominada de fase de treinamento) e prática, ambas buscando utilizar e ampliar as possibilidades dos princípios de aprendizagem motora^(17)^.

Atualmente, a terapia com o modelo *ReST* é indicada para crianças a partir de 5 anos de idade, que possuam o diagnóstico de AFI, variando a gravidade entre leve e grave. Inicialmente, é analisado o nível das pseudopalavras a serem elencadas para o tratamento e, se a criança possuir os pré-requisitos, é dado início à intervenção com pseudopalavras dissílabas, trissílabas ou sentenças de Cloze^(17)^.

Durante a aplicação do método de intervenção *ReST*, são realizadas as sondagens, sendo a mesma aplicada antes de iniciar o tratamento, no início da 4ª, 8ª e 12ª sessões, a fim de avaliar a aquisição, a retenção e a generalização **do ponto de vista** das classes das sondagens, sendo elas: pseudopalavras tratadas, pseudopalavras não tratadas, sentenças de Cloze com pseudopalavras de três sílabas, palavras reais e itens de controle^(17)^.

## APRESENTAÇÃO DOS CASOS CLÍNICOS

Este estudo está vinculado ao projeto de pesquisa Ambulatório de Desvio Fonológico e Apraxia, aprovado pelo Comitê de Ética da Universidade Federal de Santa Catarina (Parecer nº 35360620.9.0000.0121). Todos os participantes e seus responsáveis receberam informações sobre a pesquisa e, após concordarem em participar, assinaram o Termo de Consentimento Livre e Esclarecido (TCLE), o Termo de Assentimento e o Parecer Consubstanciado do CEP.

Participaram quatro crianças brasileiras, monolíngues, com idades entre cinco e oito anos, todas diagnosticadas com Atraso Motor de Fala (AMF). **A apresentação de quatro estudos de caso foi adotada para documentar a variabilidade individual de resposta ao ReST, possibilitando a observação de padrões de aquisição, retenção e generalização ao longo do tempo em um delineamento longitudinal exploratório.** Essa abordagem mostra-se especialmente adequada por se tratar de uma investigação inicial e pioneira no contexto brasileiro. A intervenção foi realizada pelo método Rapid Syllable Transition Treatment (ReST), em formato de teleatendimento síncrono, com frequência de duas sessões semanais ao longo de seis semanas. **O teleatendimento foi escolhido para favorecer acesso e continuidade terapêutica, além de dialogar com evidências e recomendações recentes que apontam a viabilidade de intervenções motoras de fala em formato remoto, desde que preservados estrutura, dose e controle de *feedback*.**

Os critérios de inclusão adotados foram: assinatura do TCLE pelos responsáveis; ser falante nativo do PB; ausência de comorbidades associadas, verificada a partir da anamnese clínica detalhada e de informações fornecidas pelos pais; desempenho de linguagem compreensiva superior ao expressivo, confirmado por observação clínica e tarefas simples de compreensão; limiares auditivos dentro da normalidade; acuidade visual preservada; e idade entre quatro e dez anos.

A coleta de dados foi organizada em três etapas principais. A primeira incluiu anamnese e avaliação fonoaudiológica, realizadas por meio da Avaliação das Habilidades Práxicas Orofaciais (The Orofacial Praxis Test)^(18)^ e da Avaliação Fonológica da Criança (AFC)^(19)^. Em seguida, foi conduzida a avaliação motora da fala diagnóstica, que envolveu o Teste de Inconsistência de Fala^(20)^, a Prova de Repetição de Palavras Multissilábicas^(20)^ e a Prova de Frase Lexical^(20)^. A terceira etapa consistiu na intervenção terapêutica com o método ReST.

Para a intervenção, foram selecionadas quatro vogais e quatro consoantes, que serviram de base para a construção de uma lista de 20 pseudopalavras. Essas pseudopalavras, dissílabas ou trissílabas, foram elaboradas de acordo com o nível de dificuldade de cada criança, seguindo o protocolo de Oliveira e Oliveira^(20)^, que prevê variação da posição do acento lexical. **Destaca-se que a escolha de pseudopalavras seguiu critérios linguísticos cuidadosos (controle de estrutura e de padrão acentual), conforme recomendado para o protocolo ReST, favorecendo interpretação e replicabilidade.** As sessões foram estruturadas em duas fases: uma fase de pré-prática, na qual o terapeuta oferecia maior suporte com feedback imediato e modelagem, e uma fase de prática, na qual as pseudopalavras eram repetidas em blocos, com feedback de resultado, não imediato e aleatório, intercalados por curtos intervalos de descanso.

As sondagens foram realizadas em diferentes momentos: antes do início da terapia, após a quarta e a oitava sessões, ao final da décima segunda sessão e, posteriormente, em um momento de follow-up, quatro meses após o término da intervenção. Em cada sondagem, foram avaliadas pseudopalavras treinadas e não treinadas, frases, palavras reais e itens de controle, de modo a verificar aquisição, retenção e generalização. **As famílias receberam orientação para apoiar a realização das sessões e para manter, ao longo da semana, um ambiente favorável à prática e à estabilidade das rotinas, sem substituição do treino terapêutico estruturado do protocolo.**

Os resultados individuais são apresentados de forma quantitativa nas Tabelas 1, 2 e 3 e ilustrados graficamente nas Figuras 1 a 4.

**Tabela 1:** Dados qualitativos nominais, expondo o perfil clínico de cada sujeito da pesquisa

	**FAIXA ETÁRIA**	**SEXO**	**REGIÃO**	**GRAVIDADE**
S1	5 anos	Masculino	Sudeste	Moderadamente severa
S2	7 anos	Masculino	Sul	Moderadamente severa
S3	7 anos	Masculino	Sul	Levemente moderada
S4	8 anos	Masculino	Sudeste	Levemente moderada

**Legenda:** S1 = Sujeito 1; S2 = sujeito 2; S3 = sujeito 3; S4 = sujeito 4. Fonte: Elaborada pelas autoras

**Tabela 2:** Dados quantitativos contínuos, expondo o índice de PCC pré e pós-intervenção *ReST* de cada sujeito da pesquisa

**SUJEITOS**	**PCC PRÉ-INTERVENÇÃO**	**PCC PÓS-INTERVENÇÃO**
S1	61,95%	82,30%
S2	55,77%	69,75%
S3	80,22%	81,25%
S4	79,25%	79,71%

**Legenda:** S1 = Sujeito 1; S2 = sujeito 2; S3 = sujeito 3; S4 = sujeito 4. Fonte: Elaborada pelas autoras

**Tabela 3:** Média, mediana e valor mínimo e máximo das sondagens realizadas com todos os sujeitos da pesquisa

**VARIÁVEL**	**MÉDIA (DP)**	**MEDIANA**	**MIN.-MAX.**
**Pseudopalavras treinadas (20)**			
Pré-intervenção	12,75 (4,26)	13	8-17
5ª sessão	15,25 (4,97)	17	7-20
9ª sessão	18 (0,71)	18	17-19
12ª sessão	18 (1,22)	17,5	17-20
Pós-tardio	17,5 (2,18)	18	14-20
**Pseudopalavras não treinadas (20)**			
Pré-intervenção	7,5 (6,22)	6	1-17
5ª sessão	12,75 (2,28)	13,5	9-15
9ª sessão	15,5 (0,87)	15	15-17
12ª sessão	17 (1,73)	16	16-20
Pós-tardio	15 (2,24)	15	12-18
**Frases (20)**			
Pré-intervenção	6,75 (4,76)	5,5	2-14
5ª sessão	11,75 (2,49)	12,5	8-14
9ª sessão	14,25 (3,03)	14,5	10-18
12ª sessão	16,5 (2,50)	16,5	13-20
Pós-tardio	13,75 (2,28)	14	11-16
**Palavras reais (20)**			
Pré-intervenção	14,25 (3,34)	14,5	10-18
5ª sessão	16,75 (1,79)	17	14-19
9ª sessão	16 (2,45)	17	12-18
12ª sessão	17,25 (2,28)	17	15-20
Pós-tardio	18,5 (2,06)	19,5	15-20
**Palavras reais com processos sonoros diferentes (10)**			
Pré-intervenção	3,25 (2,77)	2	1-8
5ª sessão	3,75 (3,11)	2,5	1-9
9ª sessão	2,75 (3,63)	1	0-9
12ª sessão	4,75 (4,32)	4,5	0-10
Pós-tardio	7,25 (2,77)	7,5	4-10

Teste Mann–Whitney

**Legenda:** DP= Desvio-padrão; Min.= Valor mínimo; Max.= Valor máximo. Fonte: Elaborada pelas autoras

**Figura 1:** Gráfico expondo o desempenho da acurácia do fonema, acento, coarticulação e resultado final da produção do S1, nas sondagens realizadas ao longo do estudo

**Fonte:** Elaborada pelas autoras

**Figura 4:** Gráfico expondo o desempenho da acurácia do fonema, acento, coarticulação e resultado final da produção do S4, nas sondagens realizadas ao longo do estudo

**Fonte:** Elaborada pelas autoras

Cada figura corresponde ao desempenho de um participante, permitindo visualizar sua evolução em termos de acurácia de fonemas, acento lexical, coarticulação e resultado final da produção. Por exemplo, na Figura 1 observa-se a progressão do participante S1 ao longo das sondagens, com aumento gradual da precisão articulatória; a Figura 2 demonstra resultados semelhantes para S2; já as Figuras 3 e 4 evidenciam o desempenho dos sujeitos S3 e S4, que também apresentaram melhora consistente, embora em ritmo distinto. Todas as etapas foram conduzidas por uma mesma fonoaudióloga, assegurando a uniformidade do processo terapêutico **(o que favorece padronização), embora possa introduzir viés de avaliação, conforme discutido adiante**.

**Figura 2 gf02:** Gráfico expondo o desempenho da acurácia do fonema, acento, coarticulação e resultado final da produção do S2, nas sondagens realizadas ao longo do estudo

**Fonte:** Elaborada pelas autoras

**Figura 3 gf03:** Gráfico expondo o desempenho da acurácia do fonema, acento, coarticulação e resultado final da produção do S3, nas sondagens realizadas ao longo do estudo

**Fonte:** Elaborada pelas autoras

## DISCUSSÃO

Os resultados deste estudo de casos indicam melhora sistemática no desempenho de fala após a intervenção com o ReST, refletida no aumento dos escores de PCC^(21)^(Tabela 2) e nas progressões observadas nas sondagens de pseudopalavras treinadas e não treinadas, frases e palavras reais (Tabela 3). Nas análises individuais (Figuras 1 [Fig gf02] [Fig gf03]-4), nota-se uma curva de evolução consistente em acurácia de fonemas, acento lexical e coarticulação, com impacto direto no resultado final da produção. Ainda que S3 e S4 tenham permanecido na mesma faixa de gravidade segundo Shriberg et al.^(21)^, a tendência de ganho percentual sugere benefício clínico e aproximação do limiar de mudança funcionalmente relevante.

A **interpretação desses achados é convergente com a literatura que sustenta o ReST** como abordagem baseada em princípios de aprendizagem motora (variabilidade, prática distribuída, sequências aleatórias e manejo do *feedback* adiado), os quais favorecem retenção e generalização^(7,12)^. O uso de pseudopalavras pode ter mitigado interferências de programas motores previamente estabilizados, permitindo reorganização de planos motores e facilitação do controle prosódico (acento lexical) e segmental (acurácia de fonemas), o que se refletiu no desempenho crescente observado nas sondagens intermediárias e no *follow-up* ^(15-17)^ *.*

Comparativamente, nossos achados dialogam com Oliveira e Oliveira^(14)^, que reportaram aquisição e generalização em crianças brasileiras com AFI submetidas ao ReST, inclusive via teleatendimento. **Além disso, a literatura tem descrito resultados favoráveis do ReST em contexto presencial e discutido a viabilidade do teleatendimento em TMF, desde que sejam assegurados critérios mínimos de qualidade e padronização do procedimento.** Embora a dose do presente estudo (duas sessões semanais durante seis semanas) seja menos intensiva que os protocolos de alta frequência, a direção e a magnitude dos ganhos foram compatíveis com o padrão de melhora descrito na literatura, especialmente quando se observam os aumentos em itens não treinados e em palavras reais (Tabela 3). Esse padrão também é coerente com a análise de Bucci^(13)^ sobre intensidade/dose, sugerindo que formatos de menor frequência podem ser efetivos quando mantêm os elementos críticos de prática e *feedback* do ReST.

**Um aspecto clínico relevante é o comportamento de generalização tardia observado para palavras reais com processos sonoros diferentes (Tabela 3): os escores foram mais modestos nas sondagens intermediárias e cresceram no pós-tardio.** Esse atraso é plausível no AMF, no qual a estabilização de novos planos motores requer mais tempo e maior exposição a contextos fonotáticos variados; em outras palavras, a transferência para alvos fonológicos/prosódicos menos familiares tende a emergir depois que o controle motor básico se consolida. Do ponto de vista terapêutico, isso reforça a importância de manter práticas de variabilidade de contexto e de prosódia mesmo após o término do bloco formal de sessões, favorecendo a retenção expandida. **Adicionalmente, esses achados sustentam a recomendação de seguimentos mais longos do que quatro meses para captar trajetórias de consolidação tardia e manutenção em contextos naturalísticos (casa, escola).**

**A viabilidade do teleatendimento é outro ponto de destaque. A manutenção de ganhos em um ambiente remoto sugere boa transportabilidade do protocolo, desde que se preservem a estrutura de sessões (pré-prática/prática), a aleatorização de itens, a cadência do *feedback* e a qualidade das pistas auditivas e visuais.** Entretanto, o teleatendimento introduz potenciais confundidores, qualidade de conexão, ruído ambiental e variabilidade no suporte familiar, que devem ser monitorados e, idealmente, controlados em estudos subsequentes. **Recomenda-se, portanto, a adoção de padronizações mínimas do ambiente e de métricas simples de qualidade do sinal, com registro sistemático dessas condições ao longo do tratamento.**

Este estudo também oferece pistas sobre mecanismos específicos de mudança no AMF. Os incrementos paralelos em acurácia segmental e acento lexical (Figuras 1 [Fig gf02] [Fig gf03]-4) sugerem que o alinhamento temporal (prosódico) e espacial (articulatório) pode ter sido co-treinado de modo funcional pelo ReST, reduzindo dependência de estratégias compensatórias (ex.: uso excessivo de mandíbula). Tal achado é coerente com a hipótese de que ajustes no *“timing”* e na hierarquia de acentos atuam como organizadores globais dos gestos articulatórios em crianças com perfis motores atrasados.

É importante destacar o valor da avaliação qualitativa dos resultados neste estudo. O acompanhamento longitudinal dos desempenhos individuais (Figuras 1 [Fig gf02] [Fig gf03]-4) permitiu observar padrões de progressão em cada participante, incluindo a melhora gradual na acurácia fonêmica, no acento lexical e na coarticulação. Esse tipo de análise é fundamental em pesquisas iniciais, principalmente em áreas ainda pouco exploradas no Brasil, como a aplicação do ReST em crianças com AMF. Ao valorizar a evolução clínica individual, é possível compreender nuances do processo terapêutico que nem sempre seriam captadas apenas por medidas estatísticas, reforçando a aplicabilidade do método na prática fonoaudiológica.

O presente estudo apresenta algumas limitações que devem ser consideradas, como o número reduzido de participantes (n=4) e a ausência de grupo controle, fatores que restringem a generalização dos achados. **De forma exploratória, não se observou um padrão único que indicasse melhor aproveitamento exclusivamente em maior ou menor gravidade, reforçando a necessidade de amostras maiores e estratificação por severidade em estudos futuros.** A coleta foi conduzida por uma única fonoaudióloga, o que garante uniformidade no procedimento, mas pode introduzir vieses relacionados à avaliação. Apesar dessas restrições, é importante destacar que este é o primeiro estudo a investigar a aplicação do ReST em crianças com AMF no Brasil, trazendo evidências iniciais relevantes e abrindo caminho para novas pesquisas controladas e com amostras ampliadas.

Para fortalecer a evidência, estudos futuros devem adotar delineamentos controlados (idealmente ensaios clínicos randomizados) com amostras maiores e **estratificação por gravidade do AMF**. **Recomenda-se investigar moderadores (idade, severidade, perfil prosódico) e mediadores (adesão, distribuição de prática, frequência de *feedback*) para elucidar quem mais se beneficia do ReST e em quais condições de dose. Além da ampliação amostral, recomenda-se padronizar procedimentos e condições de teleatendimento, incluir cegamento de avaliadores e ampliar o período de seguimento para avaliar manutenção e generalização em contextos comunicativos naturalísticos.**

No âmbito do teleatendimento, padronizações mínimas de ambiente e métricas de qualidade de sinal podem reduzir a variabilidade e aumentar a reprodutibilidade. Por fim, seguimentos mais longos que quatro meses podem captar trajetórias de consolidação tardia e manutenção de generalização para contextos comunicativos naturalísticos (escola, casa).

## COMENTÁRIOS FINAIS

Este estudo mostrou que a aplicação do método ReST em crianças brasileiras com Atraso Motor de Fala resultou em ganhos consistentes na acurácia fonêmica, no acento lexical e na coarticulação, além de favorecer aquisição, retenção e generalização dos alvos. Esses resultados reforçam a efetividade do método, inclusive em formato de teleatendimento, ampliando as possibilidades de acesso à intervenção. **Ressalta-se, ainda, que a seleção de pseudopalavras seguiu critérios linguísticos controlados, aspecto metodológico relevante para a interpretação dos achados.**

**Apesar das limitações metodológicas, trata-se do primeiro estudo brasileiro a investigar sistematicamente a aplicação do ReST em crianças com AMF**, conferindo caráter pioneiro e relevância clínica aos achados. A análise qualitativa dos dados, aliada à progressão observada nas sondagens, oferece evidências iniciais de que o ReST pode ser incorporado como alternativa terapêutica promissora na fonoaudiologia.

Novos estudos, com amostras maiores e delineamentos mais robustos, poderão confirmar e expandir esses resultados, mas os dados apresentados já contribuem para orientar a prática clínica e para fortalecer a literatura nacional na área de TMF. **Pesquisas futuras, com delineamentos mais robustos (incluindo delineamentos controlados, estratificação por gravidade, avaliadores cegos, padronização do teleatendimento e seguimentos mais longos), poderão expandir e confirmar as evidências aqui apresentadas.**
